# Real-time *in vivo* imaging of metastatic bone tumors with a targeted near-infrared fluorophore

**DOI:** 10.18632/oncotarget.20187

**Published:** 2017-08-11

**Authors:** Wonbong Lim, HongMoon Sohn, Youngjong Ko, Mineon Park, Bora Kim, Danbi Jo, Jin Seok Jung, Dae Hyeok Yang, Jangho Kim, Ok Joon Kim, Donghwi Kim, Young Lae Moon, Jung-Joon Min, Hoon Hyun

**Affiliations:** ^1^ Department of Orthopedic Surgery, Chosun University Hospital, Gwangju 61453, South Korea; ^2^ Laboratory of Orthopedic Research, Chosun University Hospital, Gwangju 61453, South Korea; ^3^ Department of Premedical Program, School of Medicine, Chosun University, Gwangju 61452, South Korea; ^4^ Department of Biomedical Sciences, Chonnam National University Medical School, Gwangju 61469, South Korea; ^5^ Institute of Cell and Tissue Engineering, College of Medicine, The Catholic University of Korea, Seoul 06591, South Korea; ^6^ Department of Rural and Biosystems Engineering, Chonnam National University, Gwangju 61186, South Korea; ^7^ Department of Oral Pathology, School of Dentistry, Chonnam National University, Gwangju 61186, South Korea; ^8^ Department of Nuclear Medicine, Chonnam National University Medical School, Gwangju 61469, South Korea

**Keywords:** metastatic bone tumor, real-time in vivo imaging, near-infrared fluorescence, targeted fluorophore

## Abstract

Tumors of the prostate or breast are particularly likely to metastasize to the bone, and early diagnosis of metastatic bone tumors is important for designing an effective treatment strategy. Imaging modalities for the detection of bone metastasis are limited, and radiation-based techniques are commonly used. Here, we investigated the efficacy of selective near-infrared (NIR) fluorescence detection of metastatic bone tumors and its role in the detection of bone metastasis in prostate and breast cancer cell lines and in a xenograft mouse model. A targeted NIR fluorophore was used to monitor metastatic bone tumors using a NIR fluorescence imaging system in real time, enabling the diagnosis of bone metastasis *in vivo* by providing the location of the metastatic bone tumor. The NIR fluorescence imaging technique using targeted NIR contrast agents is a potential tool for the early diagnosis of bone tumors.

## INTRODUCTION

Skeletal metastases are the most common malignant bone tumors. Certain types of cancer, such as those of the prostate or breast, are particularly likely to give rise to skeletal metastases, with a prevalence of up to 70% [1]. Bone metastasis is associated with severe complications, including bone pain, impaired mobility, pathological fracture, spinal cord compression, and symptomatic hypercalcemia [2].

Early diagnosis of skeletal tumors has a major impact on the overall treatment strategy and is an important determinant of the course of illness and quality of life. In particular, the detection and evaluation of bone metastases is clinically important [3]. Bone metastases are detected by imaging studies, including anatomical visualization or assessment of metabolic turnover in the metastasis itself or in the surrounding bone [3]. Bone scintigraphy (bone scan) is the most common imaging technique by using a radionuclide for the diagnosis of bone metastasis in the clinic [4, 5]. Since radiotracer uptake depends on local blood flow, osteoblastic activity, and extraction efficiency, it is not fully satisfied for the detection of cancer that has spread to the bone and metastases that are purely osteolytic. Conditions such as fractures, degenerative arthritis, and other benign bone lesions can also cause focal uptake on the bone scan and lead to false-positive results [6]. Conventional imaging techniques based on anatomical features, such as magnetic resonance imaging (MRI) or computed tomography (CT), have limitations and poor accuracy because of the ability of metastases to disturb tissue morphology [7].

In response to this unmet clinical need, optical imaging was designed to provide real-time visualization of the surgical field, especially in combination with near-infrared (NIR; 650–900 nm) fluorophores, allowing intraoperative image-guided surgery [8–11]. Optical fluorescence imaging is a fast, inexpensive, and nonionizing modality that can provide new opportunities for effective diagnostic evaluation of bone diseases by injecting a single targeted NIR fluorophore. Bone tissue-targeting NIR fluorophore, which was introduced in 2001, typically required the covalent conjugation of a bisphosphonate or other bone-targeting ligand to a NIR fluorophore, creating a bifunctional molecule for biomedical imaging [12, 13].

Recently, we have developed bone tissue-specific NIR fluorophore (P800SO3) that exhibit bone-tissue specific uptake by the virtue of structure-inherent targeting, performing both targeting and imaging simultaneously [14, 15]. We therefore hypothesized that the selective fluorescence detection of bone tumors by using P800SO3 could provide diagnostic assessment in bone metastases. In addition, noninvasive NIR fluorescence imaging using cell tracking agent, such as ESNF13, has been used to monitor the location of inoculated cancer cells *in vivo*. The lipophilic NIR cyanine fluorophore, ESNF13, previously developed for longitudinal monitoring of cell proliferation and differentiation with low cytotoxicity, high optical properties and low background outside cells. Unlike other tracking techniques, ESNF13 requires facile and simple procedures for intracellular trafficking [16]. Thus, the goal of this study was to visualize metastatic bone tumors in real-time that support the intraoperative image-guided surgery.

## RESULTS

### Binding affinity of bone tissue-specific NIR fluorophore in osteoblast and osteoclast differentiation

**Figure 1:**
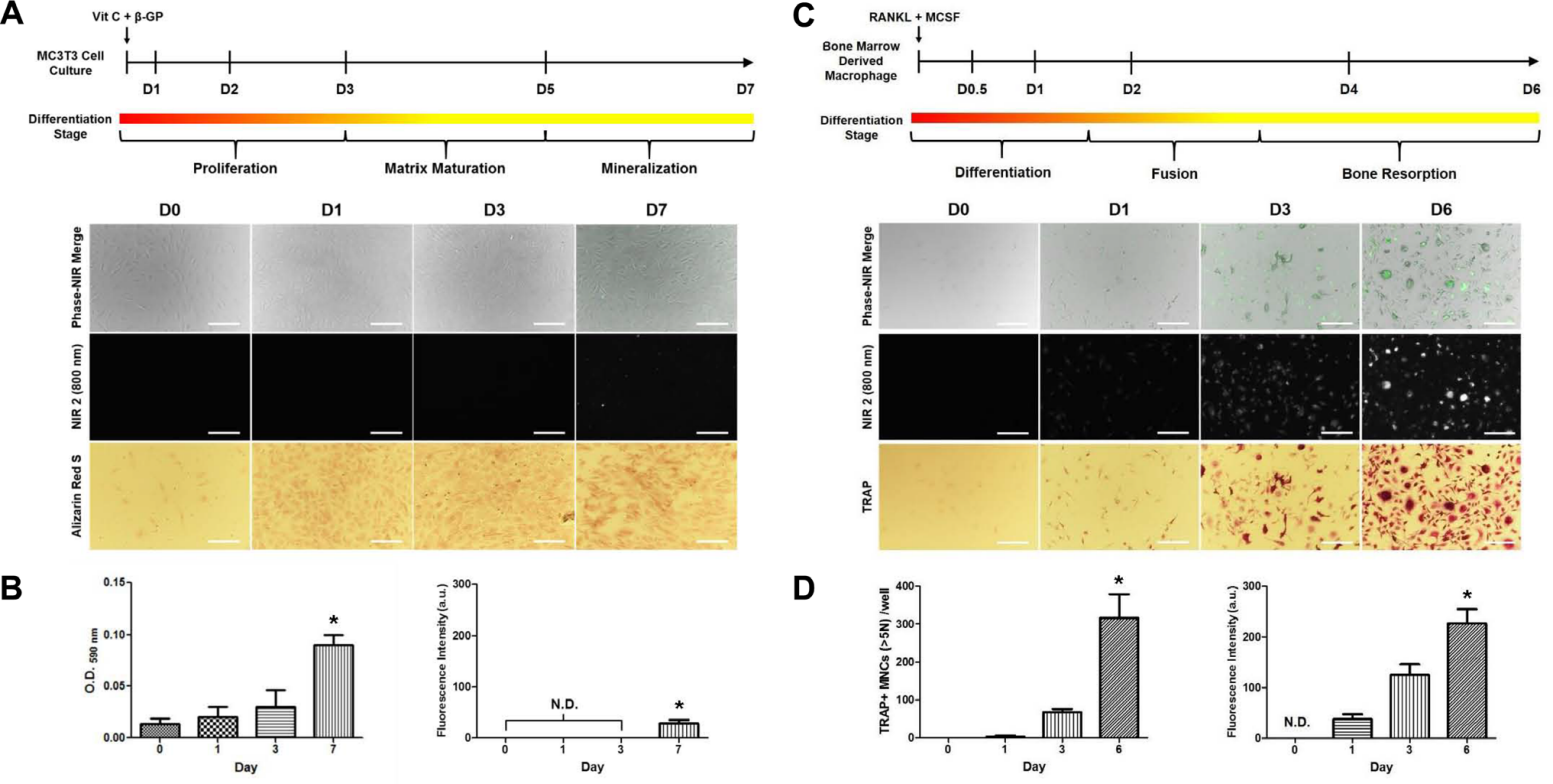
Static cell-binding assay of the specificity of the bone tissue-specific NIR fluorophore (P800SO3) and Alizarin Red S and TRAP staining at each stage of osteoblast **(A, B)** and osteoclast **(C, D)** differentiation. NIR images of each cell line tested at a concentration of 2 µM. Scale bars = 100 µm. Green pseudo-color was used for 800 nm NIR images. All NIR fluorescence images have identical exposure times and normalizations. N.D., not detected. Statistical analyses are expressed as the mean ± s.d. (*P < 0.05).

Figure 1 shows the binding affinity of P800SO3 between osteoblast and osteoclast differentiation within a week. MC3T3 osteoblast-like cell differentiation was induced by treatment with vitamin C and β-glycophosphate, and the NIR fluorophore was respectively treated at each stage of differentiation. P800SO3 binding was begun at day 7 during the mineralization stage of osteoblast differentiation, and mineralization was confirmed by Alizarin Red S staining (Figure 1A). Mineralization of MC3T3 cells was gradually increased and P800SO3 fluorescence was slightly detected at day 7 (Figure 1B). The P800SO3 was also tested for the osteoclast differentiation of bone marrow-derived macrophages (BMMs) induced by RANKL and M-CSF treatments [17]. Importantly, P800SO3 fluorescence was detected at 24 h after induction, and tartrate-resistant phosphatase (TRAP)-positive cells and fluorescence signals significantly increased at 3 and 6 days (Figure 1C). The number of mature osteoclasts with TRAP-stained multinuclear cells and NIR fluorescence intensities simultaneously increased after 24 h of RANKL treatment (Figure 1D).

### Targeted NIR imaging of skeletal tumor progression

**Figure 2:**
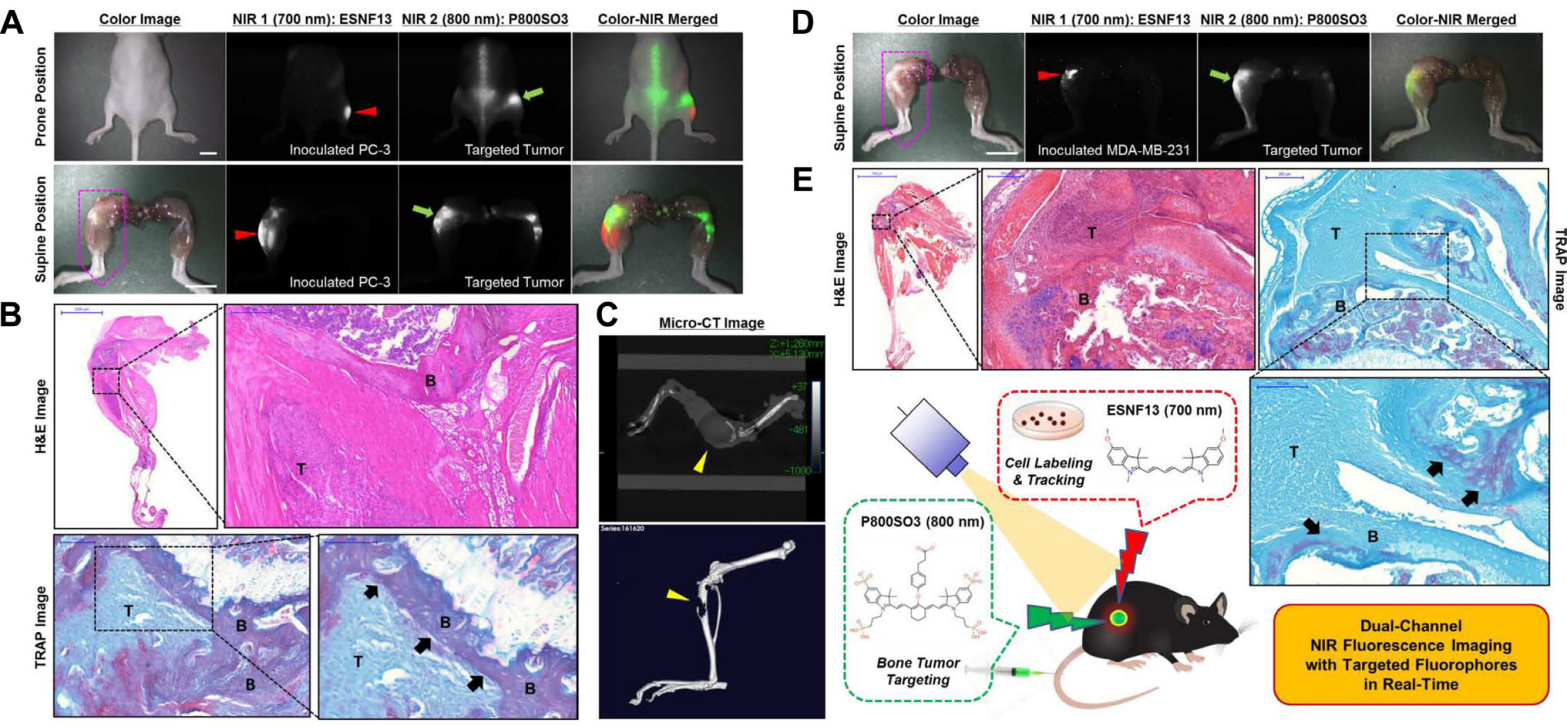
Real-time dual-channel intraoperative imaging of inoculated cancer cells and metastatic bone tumors in mice **(A, D)** Simultaneous *in vivo* NIR imaging using ESNF13-labeled PC-3 and MDA-MB-231 cells for NIR 1 channel (red arrowheads) and P800SO3-targeted tumors for NIR 2 channel (green arrows). PC-3 and MDA-MB-231 cells were intratibially inoculated into different groups of mice 3 weeks prior to imaging, followed by 10 nmol P800SO3 injected into each mouse model 4 h before imaging. Scale bars = 1 cm. Images are representative of three independent experiments. Red and green pseudo-colors were used for 700 nm and 800 nm NIR fluorescence, respectively. **(C)** Micro-CT imaging of PC-3 metastatic bone tumor in mice. The tumor site is indicated by yellow arrowheads. **(B, E)** H&E and TRAP staining of resected tumor tissues from (A) and (D) indicated by pink dots, respectively. Abbreviations: B, bone; T, tumor. All NIR images for each condition have identical exposure times and normalizations.

For real-time NIR imaging of P800SO3 during skeletal tumor progression, PC-3 or MDA-MB-231 cells stained with ESNF13 were directly inoculated into the tibia of athymic nude mice and monitored the growth of established prostate or breast tumors in the bone environment. PC-3 prostate cancer cells were observed under the 700 nm channel (NIR 1); a localized fluorescence signal was detected in the lateral tibia of the mice, and a stronger fluorescence signal of P800SO3 was detected under the 800 nm channel (NIR 2) around the left knee joint in supine position compared with the fluorescence signal in the right knee joint (Figure 2A). Fluorescence intensity of P800SO3 in the left knee joint was gradually increased 24 h post-injection, and osteolytic lesion of bone tumor was monitored in real-time during surgery. Histological analysis by H&E and TRAP staining confirmed the tumor mass and osteolytic bone lesions in the same regions as the ESNF13 and P800SO3, respectively (Figure 2B). Tumor mass was not located at the inside of bone marrow and separated from the bone. TRAP staining detected osteolytic lesions distributed broadly in the intact margin of tumors, which was confirmed with the matching regions of the P800SO3 fluorescence. It was considered that increased osteoclast activation led the osteolytic bone defect and tumor cells were leaked out from bone marrow resulted in formation of tumor mass. Taken together, tumor mass labelled with ESNF13 and osteolytic bone lesion targeted by P800SO3 were represented, separately. Although Micro-CT imaging showed apparent osteolytic bone lesion around proximal tibia, it is difficult to confirm the exact location of tumor lesions (Figure 2C). MDA-MB-231 breast cancer cells were also inoculated into the tibia, and P800SO3 fluorescence was intravenously injected after skeletal tumor progression. Expectedly, MDA-MB-231 cells were observed at the proximal tibia and distal femur, and a strong P800SO3 fluorescence was localized at the lateral tibia (Figure 2D). Similar to the observation in PC-3 injected tibia, the tumor mass and osteolytic bone lesions confirmed by H&E and TRAP staining were located in the same regions indicated by the ESNF13 and P800SO3 fluorescences, respectively (Figure 2E).

### Targeted NIR imaging of metastatic bone tumor progression

**Figure 3:**
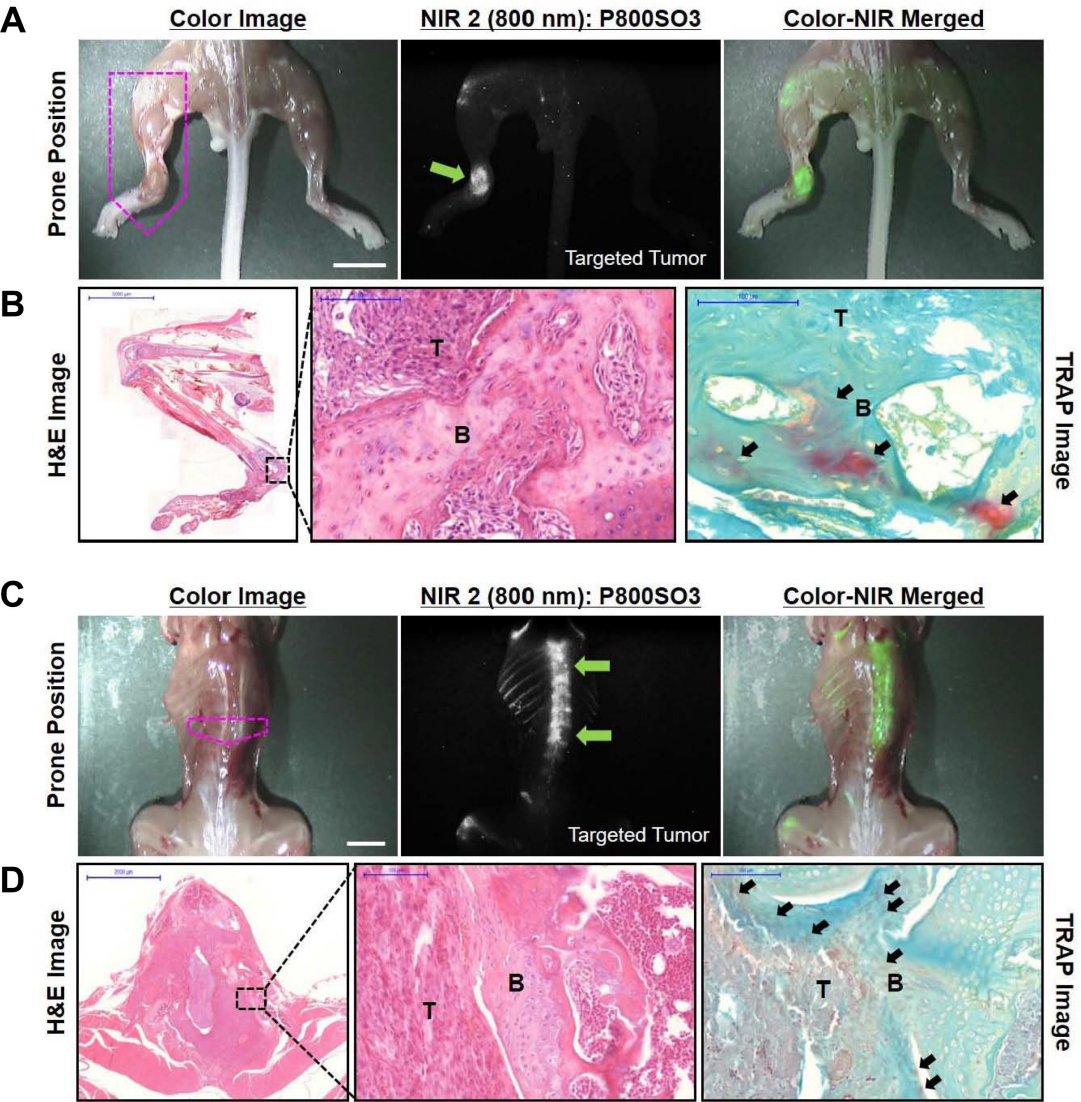
Real-time intraoperative imaging of metastatic bone tumors in mice **(A, C)** *In vivo* NIR imaging of targeted bone tumors (green arrows) under the 800 nm NIR 2 channel using P800SO3. PC-3 in a and MDA-MB-231 in c were intracardially inoculated into separate groups of mice 5 weeks prior to imaging, followed by 10 nmol P800SO3 injected into each mouse model at 24 h before imaging. Scale bars = 1 cm. Images are representative of three independent experiments. Green pseudo-color was used for 800 nm NIR fluorescence. **(B, D)** H&E and TRAP staining of resected tumor tissues from (A) and (C) indicated by pink dots, respectively. Abbreviations: B, bone; T, tumor. All NIR images for each condition have identical exposure times and normalizations.

Intracardiac injection of the mammary cancer cell lines PC-3 and MDA-MB-231, derived from a spontaneous mammary tumor occurring in the BL-6/Nu mouse, resulted in the development of metastases in the bone, as evidenced by histological analysis. The P800SO3 fluorescence was significantly observed in the hind limb of mice inoculated with PC-3 cells (Figure 3A), and bone metastasis was confirmed with predominantly osteolytic lesions, as determined by H&E and TRAP staining (Figure 3B). In addition, the intracardiac injection of MDA-MB-231 cells caused bone metastasis to the lower thoracic and upper lumbar spine. The higher fluorescence signal was also detected in the lower thoracic vertebrae, and an apparent margin between the bright field and dark area was observed at the upper lumbar spine (Figure 3C). H&E staining showed that tumors were located in the same lesions indicated by the dark areas in multiple vertebrae and involved surrounding muscle (Figure 3D). Tumor growth in the spine led to partial replacement of the bone marrow by tumor cells, and invasion progressed to the spinal canal and the intact spinal cord. Areas of active osteolysis were detected adjacent to trabecular bone surfaces at the margin of tumors by TRAP staining and matched to the increased NIR fluorescence.

## DISCUSSION

Bone is a common site of distant metastasis in prostate and breast cancers. Despite advances in our understanding of the basic molecular biology of bone metastasis, early diagnosis and disease prognosis remain important challenges because of the lack of targeted contrast agents, and bone metastasis remains a devastating complication of advanced cancer. In the last decade, bone scintigraphy became the most widely used radionuclide technique for the assessment of skeletal metastasis. However, bone scintigraphy provides non-specific images and multiple benign osseous lesions, such as eosinophilic granuloma, fibrous dysplasia and enchondroma, are difficult to distinguish from bone metastasis. Furthermore, bone scintigraphy typically assesses osteoblastic processes rather than tumor proliferation, which can lead to false-negative results, because primarily osteolytic lesions with limited reactive osteoblastic activity, such as renal cell carcinoma metastases, show low or absent tracer accumulation.

To solve this problem, optical imaging techniques that involve contrast agents targeted to skeletal tumors were developed to provide novel information on how tumor cells invade, spread, and proliferate in bone metastasis. Previously, P800SO3 was reported to show the strong binding in both calcium hydroxyapatite (HA) and calcium phosphate (CP) as bone matrix components resulting in bone tissue-specific targeting compared to that of a conventional bisphosphonate conjugate binding to HA only. That is, P800SO3 could highlight bone resorption where CP is released, as well as neo-ossification where HA is formed. Because activated osteoclasts were involved in the direct uptake of P800SO3, we hypothesized that the fluorescence intensity in the osteoclast-activated lesion, such as in skeletal metastatic tumor progression, could be highlighted strongly. In the present study, P800SO3 uptake was higher in TRAP-positive osteoclasts than in mineralized osteoblasts, suggesting that the increased P800SO3 fluorescence signals indicated an osteolytic lesion in bone tissue.

Tumor invasion during skeletal metastatic progression shows osteogenetic and osteolytic features. In particular, metastatic breast cancer cells in the bone marrow hijack signals from the normal bone remodeling process and promote bone degradation [18]. Bone degradation is caused by osteoclasts, which have a unique ability to dissolve bone mineral and degrade the bone matrix. These features make them a predominant actor in bone metastasis formation. This is the reason why anti-resorptive drugs such as bisphosphonates and denosumab, a human monoclonal antibody against osteoclasts, are used in the clinic as palliative treatment to interfere with bone resorption. As shown in this study, P800SO3 bound strongly to active osteoclasts, similar to bisphosphonate, in PC-3 and MDA-MB-231 skeletal tumors induced by intratibial or intracardiac inoculation. Tumor invasion from the bone marrow to bone and muscle induced by intratibial inoculation of PC-3 or MDA-MB-231 cells was readily detected by monitoring osteolytic lesions after a single injection of P800SO3.

The vertebrae, pelvis, ribs, and ends of long bones are preferred destinations of metastases because of their high red marrow content [19]. Metastatic deposits were easily detected and quantified in the present study, confirmed with H&E staining. Although the detection of tumors by a NIR fluorescent protein in deep organs was demonstrated previously by Filonov et al., there are no data on the use of such a tool for visualization of metastatic bone tumors [20].

Considering that benign osseous lesions and bone metastases often show similar imaging features, NIR fluorescence imaging can be used to distinguish between them in equivocal cases of lesion in the bone metastatic tumor. Since bone scintigraphy provides the less accurate diagnosis of osteolytic bone metastatic tumor than osteogenic, the target-specific NIR fluorescence imaging could be a great alternative tool for diagnosis of bone metastasis. Moreover, real-time fluorescence detection of osteolytic lesion on metastatic tumor could help surgeons for intraoperative image-guided surgery. The future of imaging bone metastasis using the targeted NIR fluorophore will likely involve the development of an array of new tumor-specific tracers that will greatly increase diagnostic accuracy.

## MATERIALS AND METHODS

### NIR fluorophores

ESNF13 and P800SO3 were synthesized as described previously [14, 16] and dissolved in dimethyl sulfoxide (DMSO) to generate 10 mM stock solutions. Cells were labeled with ESNF13 at a concentration of 2 μM in 24-well plates (5 × 10^4^ cells per well), and P800SO3 was injected intravenously into 25 g athymic nude mice (10 nmol, 0.4 mg/kg) prior to imaging.

### Cell culture and animals

PC-3 and MDA-MB-231 cells were maintained in RPMI 1640 medium (Gibco-BRL, Paisley, UK) supplemented with 10% fetal bovine serum (FBS; Gibco-BRL, Paisley, UK) and Antibiotic Antimycotic Solution (100 units/mL penicillin, 100 μg/mL streptomycin, 0.25 μg/mL amphotericin B; Welgene, Daegu, South Korea) in humidified 5% CO_2_/95% air at 37°C. MC3T3-E1, a mouse osteoblastic cell line, was maintained in growth medium (α-MEM; Gibco-BRL, Paisley, UK) supplemented with 10% FBS and Antibiotic Antimycotic Solution. Cells were cultured for 24 h at a density of 1 × 10^5^ cells/mL in 10 cm culture dishes or 24-well plates (Corning Inc., Corning, NY, USA). After reaching confluence, cells were cultured in differentiation medium supplemented with 50 mg/mL ascorbic acid and 10 mM β-glycerophosphate (Sigma-Aldrich, St. Louis, MO, USA), and the medium was changed every 3 days. The 5-week-old male athymic nude mice (BL-6/Nu; Orient Bio Co. LTD, Seoul, South Korea) were housed under controlled conditions of light and fed ad libitum. All experimental procedures involving animals were performed in compliance with institutional and governmental requirements and approved by the Institutional Animal Care and Use Committee (CIACUC2017-A0002) of Chosun University, Gwangju, South Korea.

### BMM cell culture

Bone marrow cells were collected by flushing the tibiae and femurs from mice with α-MEM medium (Gibco-BRL, Paisley, UK), as previously described [21]. After the red blood cells were depleted with ACK (ammonium-chloride-potassium) lysis buffer (0.01 mM EDTA, 0.011 M KHCO_3_, and 0.155 M NH_4_Cl, pH 7.3), the remaining cells were suspended in complete α-MEM supplemented with 10% (v/v) FBS, 100 U/mL penicillin, and 100 μg/mL streptomycin. The cells were then incubated for 24 h in the presence of 10 ng/mL M-CSF (Cell Guidance Systems Ltd., Cambridge, UK). Non-adherent cells were collected and cultured with 30 ng/mL M-CSF for 3 days to generate BMMs, and BMMs (50,000 cells/cm^2^) were cultured with 30 ng/mL M-CSF and 37.5 ng/mL RANKL (Cell Guidance Systems Ltd., Cambridge, UK) for 3 days in complete α-MEM to generate osteoclasts.

### *In vitro* cell assay

NIR imaging of osteoblasts and osteoclasts was performed on a 4-filter set Nikon Eclipse Ti-U inverted microscope system. The microscope was equipped with a 100 W halogen lamp, NIR-compatible optics, and a NIR-compatible 10× Plan Fluor objective lens (Nikon, Seoul, South Korea). Image acquisition and analysis were performed using NIS-Elements Basic Research software (Nikon, Seoul, South Korea). Two NIR filter sets composed of 650 ± 22 nm and 750 ± 25 nm excitation filters, 675 nm and 785 nm dichroic mirrors, and 710 ± 25 nm and 810 ± 20 nm emission filters were respectively used to detect P800SO3 signals in the samples. All NIR fluorescence images had identical exposure times and normalization.

### Quantitative analysis

At each time point, the fluorescence intensity was quantified using ImageJ version 1.51 k. All NIR fluorescence images for a particular fluorophore were normalized identically for all conditions of an experiment. At least three sets of data were analyzed at each time point. Statistical analysis was performed using one-way ANOVA. Results were presented as the mean ± standard deviation (s.d.), and curve fitting was performed using Prism version 4.0a software (GraphPad, San Diego, CA, USA).

### Alizarin Red S staining

At the end of the experimental period (at day 7), mineralized bone nodules were observed by staining with Alizarin Red S (Sigma-Aldrich, St. Louis, MO, USA). Cells were washed, fixed with 95% ethanol at 4°C for 15 min, and then stained with 2% Alizarin Red S solution for 10 min. Unbound stain from the culture was removed, and images of red-stained mineralized matrix surfaces were captured using a Nikon Eclipse Ti-U inverted microscope system (Nikon, Seoul, South Korea). For quantification of staining, 800 μL of 10% acetic acid (Sigma-Aldrich, St. Louis, MO, USA) was added to each well, and the plate was incubated at 25°C for 30 min. The cells were scraped and transferred to a 1.5 mL microcentrifuge. After vortexing for 30 s, the slurry was overlaid with 500 μL of mineral oil (Sigma-Aldrich, St. Louis, MO, USA), heated to exactly 85°C for 10 min, and transferred to ice for 5 min. The slurry was then centrifuged at 20,000 × *g* for 15 min and 500 μL of the supernatant moved to a new microcentrifuge tube. Then 200 μL of 10% ammonium hydroxide (Sigma-Aldrich, St. Louis, MO, USA) was added and read in 96-wells plate at 405 nm by an ELISA reader.

### TRAP staining

BMMs were treated with M-CSF and RANKL for 3 days, and the generated osteoclasts were stained for TRAP by incubation in a solution containing fast red violet LB salt and naphthol AS-MX phosphate in N,N-dimethylformamide (Sigma-Aldrich, St. Louis, MO, USA). The solution was prepared according to the manufacturer’s instructions. Osteoclasts were first washed with phosphate buffered saline (PBS) and then fixed with 10% formalin for 5 min. After three washes with distilled water, the fixed cells were stained with fast red violet/naphthol AS-MX phosphate solution for 30–40 min and observed under a Nikon Eclipse Ti-U inverted microscope system (Nikon, Seoul, South Korea). Images of the stained cells were captured using a digital camera attached to the microscope. Multinuclear cells were manually counted.

### Intratibial xenograft model

Intratibial injection of prostate cancer cells followed by *ex vivo* imaging was used to examine the ability of prostate and breast cancer cells from home to bone. Severe combined immunodeficient male mice (5–6 weeks old) were anesthetized with isoflurane gas, and PC-3 or MDA-MB-231 cells (1 × 10^5^ cells per mouse) were injected into the right tibia. Briefly, both knee joints were shaved and cells in 20 μL DMEM were injected using a 100 μL Hamilton type syringe with a 27-gauge needle. The needle was inserted into the intra condylar notch of the femur using a spinning motion. Cells were injected into the medullary space (approximately 0.3 cm proximal to the epiphy seal plate). To control for surgical effects, 5 μL DMEM was injected into the contralateral limb. Following injection, 0.1 mg/kg buprenorphine was injected subcutaneously to minimize post-procedure pain and animals were returned to cages for recovery. *Ex vivo* images of tumor-bearing tissues excised from the mice at necropsy were obtained at 3 weeks.

### Intracardiac xenograft model of bone metastasis

For intracardiac injection, PC-3 and MDA-MB-231 cells (3 × 10^5^ cells in 0.1 mL sterile PBS) were injected into the left cardiac ventricle of mice with a 25-gauge syringe, as previously described, under deep anesthesia with isoflurane (100 mg/kg body weight) solution [22]. Images of tumor-bearing tissues excised from the mice at necropsy were obtained at 5 weeks.

### Intraoperative optical imaging system

*In vivo* NIR fluorescence imaging was performed using Mini-FLARE^®^ imaging system [9] donated by Beth Israel Deaconess Medical Center (Boston, MA, USA) to Chonnam National University Hwasun Hospital (Hwasun, South Korea) for research and clinical use in 2012. Briefly, the system consists of three separate light sources of different wavelengths: a “white” LED light source, generating 26,600 lux of 400–650 nm light to illuminate the surgical field, and NIR LED light sources, generating Channel #1 (656–678 nm excitation; 689–725 nm emission; 1.08 mW/cm^2^ fluence rate) and Channel #2 (745–779 nm excitation; 800–848 nm emission; 7.70 mW/cm^2^ fluence rate). White light and NIR fluorescence images are acquired simultaneously and displayed in real time using custom designed optics and software. Fluorescence intensity was calculated using the Mini-FLARE^®^ software, which allows quantitative measurements.

### Microcomputed tomography

The right femur and tibia was dissected from each mouse, and CT imaging was performed using a Quantum GX μCT imaging system (PerkinElmer, Hopkinton, MA, USA) located at the Korea Basic Science Institute (Gwangju, South Korea). The X-ray source was set at 90 kV and 88 mA, with a field of view of 45 mm (voxel size, 90 μm; scanning time, 14 min). The CT images were represented in 3D Viewer, the software supplied with the Quantum GX system. Following scanning, image segmentation was performed using Analyze software (AnalyzeDirect, Overland Park, KS, USA). Briefly, segmentation of the leg was performed using both semi-automatic and manual tools (e.g., object extraction, region growing, and objector separator) using the Volume Edit tool. Subsequently, a 3D rendering of the leg was generated using the ROI tool.

### Histologic analysis

Tissues were fixed in cold 4% paraformaldehyde. Bone tissue was first decalcified using a sodium citrate solution before processing into histologic slides. The decalcified bones were cut at the midpoint and embedded in paraffin blocks. The serial paraffin sections were stained with H&E and TRAP. The stained tissues were captured by Microscope slide scanner (3D-HISTECH Ltd., Budapest, Hungary).

### Statistical analysis

All analyses were performed using GraphPad Prism Ver. 6.0 (GraphPad Software Inc. San Diego, CA, USA). All data were expressed as mean ± s.d. with statistical significance considered with *P* < 0.05. Statistical comparisons between 0 day and others were determined using one-way analysis of variance (ANOVA) with Tukey’s post-hoc test, respectively. Data were analyzed using SPSS version 20.0 software for Windows (SPSS, Chicago, IL, USA).
